# The response of *Synechococcus sp*. PCC 7002 to micro-/nano polyethylene particles - Investigation of a key anthropogenic stressor

**DOI:** 10.1371/journal.pone.0232745

**Published:** 2020-07-01

**Authors:** Mary C. Machado, Gina V. Vimbela, Tania T. Silva-Oliveira, Arijit Bose, Anubhav Tripathi

**Affiliations:** 1 Center for Biomedical Engineering, School of Engineering, Brown University, Providence, Rhode Island, United States of America; 2 University of Rhode Island, Kingston, Rhode Island, United States of America; University of California, Merced, UNITED STATES

## Abstract

Microplastics or plastic particles less than 5 mm in size are a ubiquitous and damaging pollutant in the marine environment. However, the interactions between these plastic particles and marine microorganisms are just starting to be understood. The objective of this study was to measure the responses of a characteristic marine organism (*Synechococcus sp*. PCC 7002) to an anthropogenic stressor (polyethelene nanoparticles and microparticles) using molecular techniques. This investigation showed that polyethylene microparticles and nanoparticles have genetic, enzymatic and morphological effects on *Synechococcus sp*. PCC 7002. An RT-PCR analysis showed increases in the expression of esterase and hydrolase genes at 5 days of exposure to polyethylene nanoparticles and at 10 days of exposure to polyethylene microparticles. A qualitative enzymatic assay also showed esterase activity in nanoparticle exposed samples. Cryo-scanning electron microscopy was used to assess morphological changes in exopolymer formation resulting from exposure to polyethylene microparticles and nanoparticles. The data from this paper suggests that microplastic and nanoplastics could be key microbial stressors and should be investigated in further detail.

## Introduction

Microplastics, or plastic particles less than 5 millimeters, pose a significant challenge to the health of marine habitats across the globe. [1] Studies suggest microplastic is one of the most abundant forms of solid waste on earth, accounting for 92% of plastic waste on the ocean’s surface. The world’s oceans are thought to contain somewhere between 93 and 236 metric tons of microplastic particles with nearly 8 tons added each year. Polyethylene is one of the most manufactured polymers [2], and forms the majority of the microplastic particles on the ocean’s surface in many areas [3], [4]. The impacts of these long half-life plastics on the ocean environment are just starting to be understood. [5] The study of nanoplastics, or particles smaller than a few micrometers in size, is even less developed. [6], [7]

Although microplastics have been shown to be distributed globally throughout the oceans, scientists lack a extensive understanding of the concentrations, cycling and fate of these particles, impeding progress on understanding the full ecotoxicological effects of these particles on the marine environment. [8] To date, research on microplastics has been focused on the species level and centered mainly on species that ingest the particles directly.

One pressing area of investigation includes the interaction of microplastics with marine microorganisms. The diversity of microplastic-associated microorganisms is starting to be understood. Studies suggest that the shape, size and composition of microplastic can have large effects on the type of bacteria colonizing the surface of the plastic [9]. These factors can also influence biofilm adhesion and development on these surfaces. [10], [11] Recent literature also suggests that plastics exposed to the marine environment may be photo-oxidized at a rate much faster previously thought allowing for adhesion and perhaps further degradation by marine organisms. [12] However, the ecotoxicological effects of microplastics and nanoplastics on microorganisms remain largely unknown. Hydrocarbon degrading species could hold great promise for microplastic degradation efforts, however few studies have focused on the interactions of possible plastic-degrading bacteria like *Synechococcus sp*. *pcc 7002* with microplastic and nanoplastic. Members of the genus *Synechococcus* including *Synechococcus sp*. *pcc 7002* are found in high concentrations in coastal waters. [13] In addition, this unicellular cyanobacteria is one of the few species to have a fully sequenced genome, making it an ideal initial candidate for gene expression analysis. [14]

New techniques are needed to assess and analyze the effects of plastic on marine bacteria growth and biofilm formation. One such tool is quantitative real time PCR (qPCR) which is an accurate methodology for analyzing difference in the functional status of a cell. [15] Another sensitive and powerful tool for analyzing the effects of environmental stressors is reverse transcription- polymerase chain reaction (RT-PCR), a technique where RNA is first reverse transcribed into complementary DNA and then this DNA is amplified using qPCR. [16] RT-PCR permits gene expression analysis from small amounts of RNA, and can be done for a large number of genes. [16] However, the adaptation of molecular tools such as RT-PCR into environmental analysis has been slow [17] and although expression analysis of microplastic ingestion is fairly common within the literature, this innovative technique not yet been applied to the investigation of microplastic interactions with marine microorganisms.

The objective of this study was to measure changes in the viability, gene expression and morphology of a characteristic marine organism (*Synechococcus elongatus pcc 7002*) exposed to the anthropogenic stressor of polyethylene nanoparticles and microparticles. To accomplish this, we used gene expression analysis and cryo-SEM analysis to examine the reaction of *Synechococcus elongatus pcc 7002* to these stressors in detail.

## Methods

### Experimental sample preparation

#### Stock cell culture conditions and growth monitoring

*Synechococcus sp*. *PCC 7002* was acquired from the American Type Culture Collection (ATCC). Starter cultures were transferred from a bubbled stock culture and grown in 70 mL of A+ media (UTEX Culture Collection of Algae, Austin, Texas) in baffled flasks on an orbital shaker Innova 4080 shaking incubator (Eppendorf, New York, NY) at 30°C and 100 rpm. A+ media was formulated according to the UTEX algal media culture recipe with a pH between 6 and 8 and a salinity of 18 ppt. [18] Cultures were exposed to a diurnal lighting cycle of 12 hours. Cultures were grown to a pellet wet weight of 0.5 mg.

**Fig 1:**
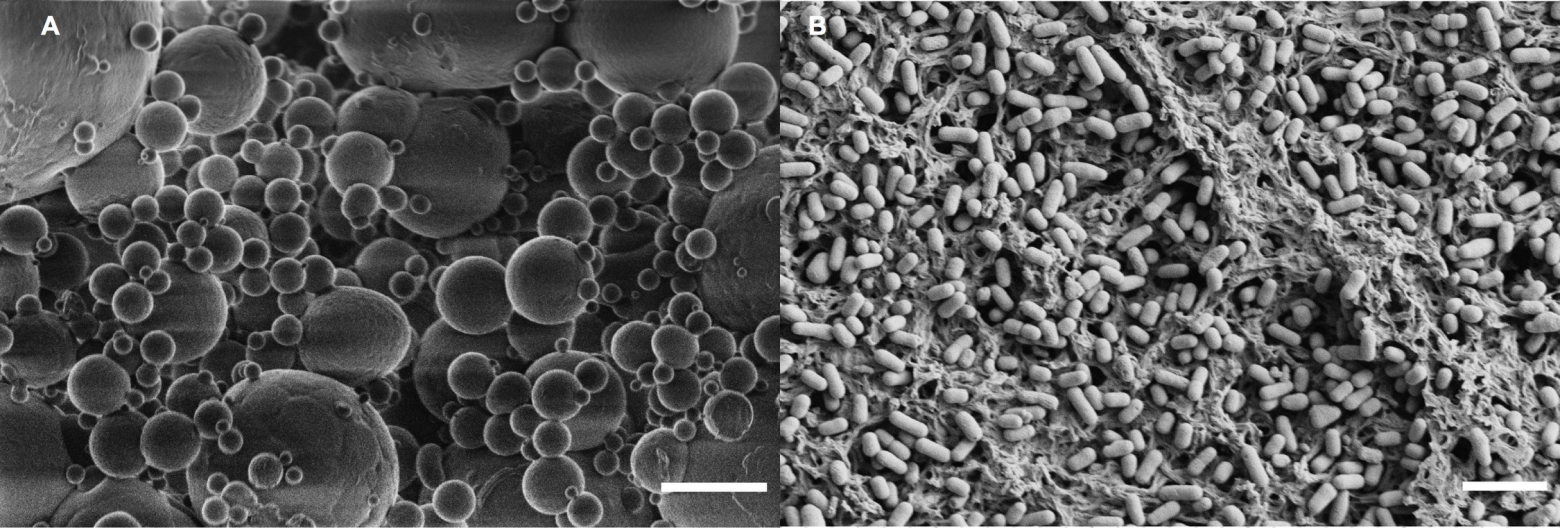
SEM of experimental materials. A) polyethylene nanospheres (200–9900 nm) B) *Synechococcus sp*. *PCC 7002* Scale bars = 5 μm.

*Polyethylene particle suspension*. Prior to sample preparation, polyethylene particulates (Fig 1) were suspended in Tween20 (Sigma-Aldrich, St. Louis, MO). The suspensions were created by adding polyethylene nanospheres (200–9900 nm; density 0.953 g/cc) or clear polyethylene microspheres (1–4 μm; density 0.96 g/cc) acquired from Cospheric (Santa Barbara, CA) to a freshly made 0.1% Tween20 solution and centrifuging twice at 12000 RPM for 10 min to get particles into the solution. The mixtures were then sonicated for 30 min in a water-bath sonicator (VWR, Radnor, PA) to make a homogenous particle mixture.

*Creation of experimental samples*. To create experimental samples 3 mL of a stock cyanobacterial culture was added to each glass reaction vial. A+ media was added to this stock cyanobacterial culture, and then a 20% (w/v) suspension of polyethylene nanoparticles in 0.1% Tween20 or a 20% (w/v) suspension of polyethylene microspheres in 0.1% Tween20 was added to this final solution. The concentration of particles within the experimental setup was adjusted to 0.00050 g/mL [19], [20] to better match literature values. A negative control of 0.1% Tween20 without particles was also included in each experimental trial.

Experimental samples were incubated under fluorescent light at 30°C and under continuous shaking of 118 rpm in an Innova 4080 shaking incubator (Eppendorf, New York, NY) for 10 days.

*Viability measurements and imaging analysis*. Cell density and viability of experimental samples was measured using a direct counting method at 24-hr intervals. Briefly, cells were stained with 0.33% neutral red and measured with a Neubauer improved disposable hemocytometer (Bulldog Bio, Portsmouth, NH) via phasecontrast/brightfield microscopy (VWR, Radnor, PA). 1 mL aliquots of cell culture were extracted at day 0, 5 and 10 for cell density measurements. Briefly, each aliquot was centrifuged at 4500 rpm for 10 min and the supernatant was removed and discarded. Wet weight of cell pellets was then recorded.

Biofilm samples were fixed with 3% glutaraldehyde, and dehydrated using first an ethanol series and then by air drying with hexamethyldisilazane (HDMS) as a drying agent. Samples were imaged using a Zeiss Sigma VP field emission scanning electron microscope (Zeiss, Oberkochen, Germany).

CyroSEM images were obtained with a SIGMA VP Field Emission-Scanning Electron Microscope operated at 3kV. Samples were sputtered with platinum.

#### Qualitative hydrolytic assay for esterase/lipase

Tween marine agar plates were set up according to protocols found within the literature. [21] Briefly isolation plates were created by the addition of Tween 20 (1% final concentration) to a marine agar medium. Experimental samples from days 0, 5 and 10 were stabbed into agar surface and plates were incubated overnight at 30°C. Clearance halos were detected and measured for a period of 48 hrs.

### RNA analysis

#### RNA extraction

2 mL aliquots were extracted from experimental samples at day 0, 5 and 10 for RNA analysis. Each aliquot was centrifuged at 4500 rpm for 10 min and the supernatant was removed and discarded. Cells were then re-suspended in 300 μL of TRI reagent (Zymo Research, Irvine, CA) and homogenized in a Beadbug homogenizer (Benchmark Scientific, Edison, NJ) at 4000 rpm for 20s. Total RNA was extracted using the Direct-zol RNA miniprep plus kit (Zymo Research, Irvine, CA) following the manufacturer’s protocol. A DNAse digestion was performed on column during this procedure.

#### RNA concentration and purity analysis

RNA concentration and purity were analyzed spectrophotometrically and electrophoretically to ensure RNA integrity. RNA purity and concentration was first assessed by a NanoDrop ND-1000 UV/Vis spectrophotometer (NanoDrop Technologies, USA). Total RNA concentration (ng/uL) and absorbance rations (260/280 and 230/280) were recorded for three biological replicates of each experimental condition. RNA integrity was further assessed using the Agilent Bioanalyzer 2100 (Agilent Technologies) along with the Agilent RNA 6000 Pico Kit. The Agilent 2100 Expert Software was used to determine RIN number, to assess peak height and to quantify RNA integrity.

#### RT-PCR analysis

*Primer selection and optimization*. A set of 4 target genes and 3 references was selected from the literature for further evaluation. [22–26] Primer pairs and amplicon characteristics (name, abbreviation, primer sequence, and cyanobase ID number) are listed in Table 1. Melting temperature and GC% of the primers were calculated using OligoAnalyzer (Integrated DNA technologies, Leuven, Belgium). Primer sequences were analyzed for specificity using Blast.

**Table 1:** Reference genes [22] and potential hydroloases and esterases were identified from the literature. [23], [24], [25] Evaluation of a metacapase (p-20 domain protein) [26] was also included within this analysis.

Name	Proposed function	Gene ID	Forward Sequence	Reverse Sequence
rimM	16S rRNA processing protein: synthesis and modification	SYNPCC7002_A1245	GATCGCCCCGAACTCGAAGC	TTCTGGTTGGCATCGGTGACTTC
secA	Part of the Sec protein translocase complex	SYNPCC7002_A1259	GCCGAAATGAGAACCGGGGAAG	GAAACGGTGTACCTGCCCCATC
rbcL	Biological Process: carbon utilization by fixation of carbon dioxide	SYNPCC7002_A1798	GTGCATGATGATGGGAGTGCGA	CTACAAGGGTCGTTGCTA
Esterase (Est)	Esterase/lipase	SYNPCC7002_G0018	TCCCCCCAAACTTGAACC	CGCACCCTCTATCTCCAC
serine esterase (SerEst)	hydrolase activity	SYNPCC7002_A2540	CCCTGAAAATCTTCCCGTTCC	ATTTGCCAACTGCCCACC
caspase	p20 domain protein	SYNPCC7002_A0101	CCAGCCAGCGATACCAAC	TGCCCCTTTTCTCCCACC
PET Hydrolase (PETHydro)	Hypothetic polyethylene hydrolase	SYNPCC7002_A0234	TGCCGAAACGGGTCAAGTCC	GATCCGCCATCTCTGGCTCG

An additional in-depth analysis was performed to identify alpha/beta hydrolases within the *Synechococcus sp*. PCC 7002 genome using a Blastp. A highly expressed, plastic degrading alpha/beta hydrolase from *Ideonella sakaiensis* [27]was compared to the genome of several species of *Synechococcus*. This sequence similarity analysis provided sequences of good similarly (and assumed homology) across multiple species. A strain with a high sequence similarity in *Synechococcus sp*. PCC 7002 and a high E value was selected.

*RT-PCR quantitation*. Total RNA from extracted samples (1.0 ug) was reverse-transcribed using a RevertAidTM H Minus First Strand cDNA Synthesis Kit (Fermentas, Thermo Fisher Scientific, Waltham, MA) in 20-μl reaction mixture containing using random hexamers. Optional steps for G/C rich templates were added to the manufactures’ protocol. These steps included heating template RNA and primer for 5 minutes at 65˚C and increasing the temperature of incubation with reverse transcriptase to 45˚C.

Quantitative real time PCR was performed using a Biorad CFX384 Real Time PCR machine (Biorad, Hercules, CA) with SYBR Green fluorescent dye (Luna Universal qPCR Master Mix (NEB, Ipswich, MA)). The amplification profile used for this experiment was the following, 95°C for 60s, followed by 40 cycles of 95°C for 15 s and 60°C for 30s. After amplification a melting curve analysis was performed for each experimental run. Settings for this run were 60°C to 95°C, with a ramp of 0.5°C and 5 s increments.

All experiments were performed in triplicate with two technical replications for every experimental replicate. Each analysis contained a negative control (nuclease free water) and a positive control (reference DNA sample). DNA contamination after on column DNAse treatment was assessed by the inclusion of negative reverse transcription controls into RT-PCR experiments.

#### RT-PCR evaluation and statistical analysis

Fluorescence data from RT-PCR amplification was analyzed using Biorad CFX Manager Software v2.1 (BioRad). Coefficients of variation (C_q_) were determined using a multivariable, nonlinear regression model. To compensate for inter-PCR variations, normalization of the target gene was performed to an endogenous internal control. Reference genes (Table 1) were analyzed for expression stability using geNorm software. Target gene relative expression ratios (R) were expressed using the 2^−ΔΔCT^ method [28]. Gene expression analysis was carried out using CFX Manager Software (BioRad). Statistical analysis RT-PCR data from cyanobacteria samples was performed using a two-way ANOVA. The Tukey HSD test was used to evaluate the statistical significance of interactions between each factor’s levels. The two factor ANOVA to evaluate the experimental optimization had the following factors and levels: particle size (no particles added, nanometers, micrometers), time of growth (0 days, 5 days and 10 days). A p- value of 0.05 was considered to be statistically significant. Statistical analysis was performed using a the XLSTAT statistical software package (Addinsoft, New York, NY).

## Results

### Cell viability and biofilm formation

**Fig 2:**
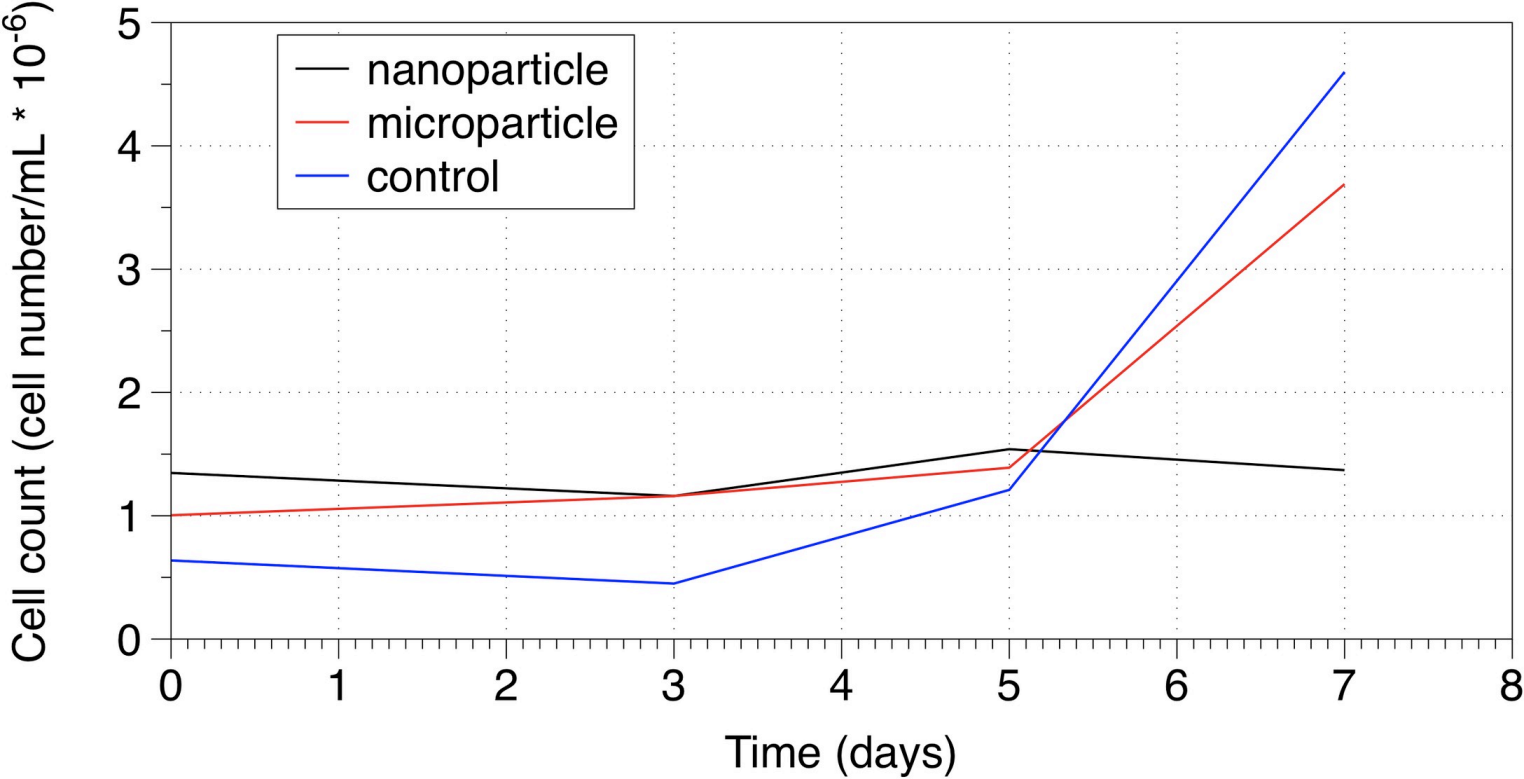
Viability analysis of *Synechococcus sp*. *PCC 7002* exposed to polyethylene nanoparticles and microparticles over a period of 7 days.

**Fig 3:**
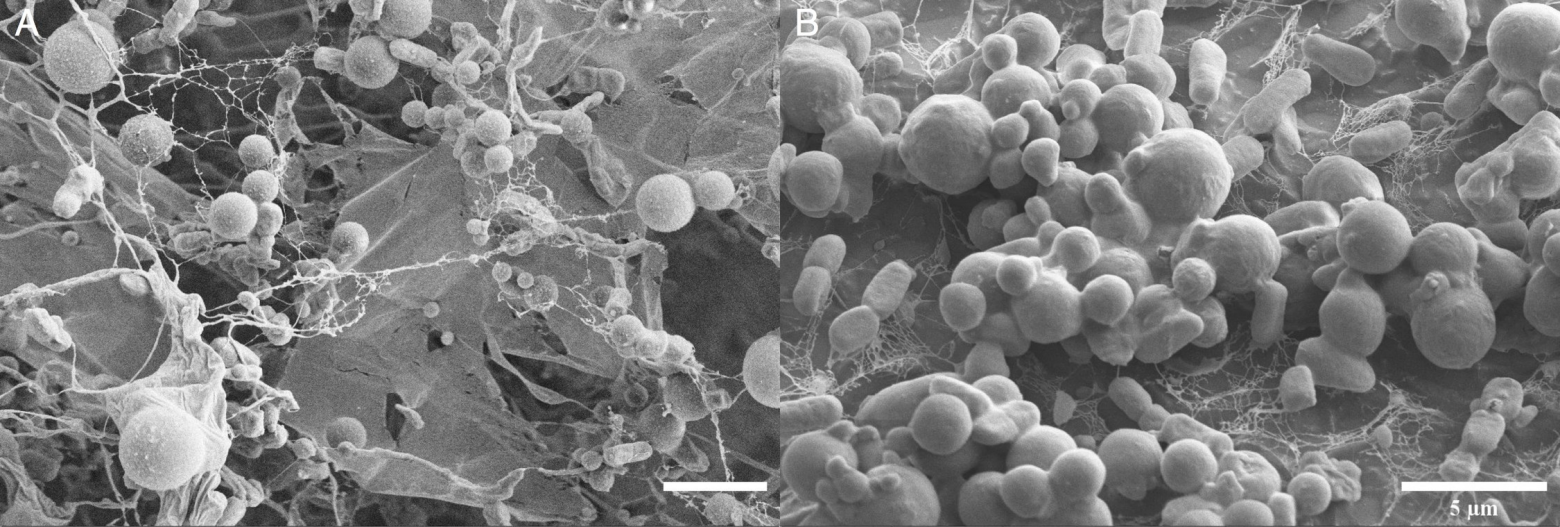
Cryo-SEM analysis of *Synechococcus sp*. *PCC 7002* on a) polyethylene nanoparticles and b) polyethylene microparticles with EPS fibril structure highlighted. Scale bars = 5 μm.

Fig 2 shows increases in cell viability within control and microparticle samples over a period of 7 days, where nanoparticle samples remained static for this time period. Cryo-SEM analysis showed the appearance of biofilm morphology including extracellular polysaccharides (EPS) fibril formation on polyethylene particle surfaces within 12 days of exposure to particulate solutions. EPS fibril structures as highlighted in Fig 3 could be seen in both polyethylene nanoparticle and polyethylene microparticle samples.

### Tween 20 plate assay

**Fig 4:**
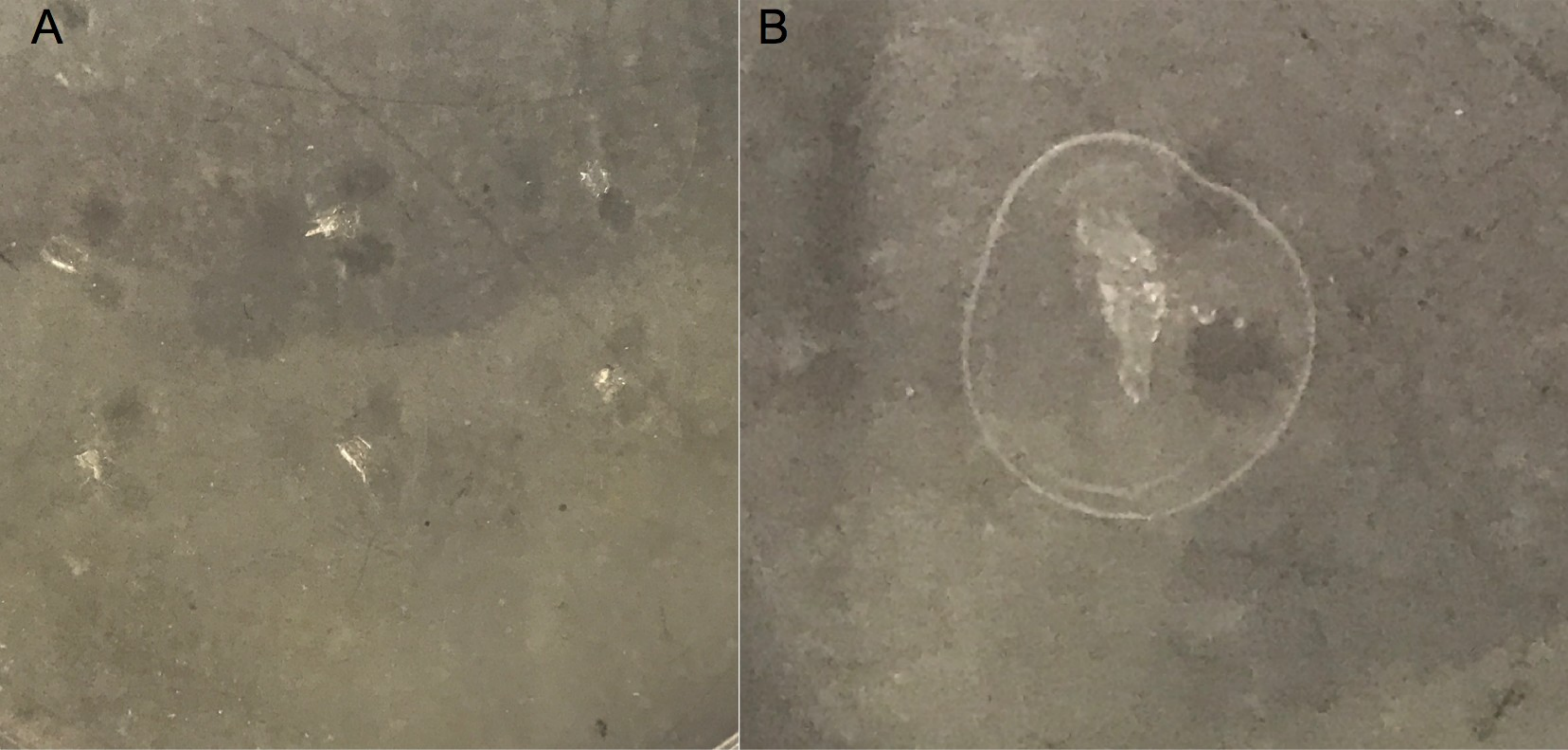
1% Tween 20 agar plate assay. Results of a 1% Tween 20 agar plate assay at 5 hours exposure to A) polyethylene microparticles B) polyethylene nanoparticles.

Qualitative screening of the esterase activity of the experimental samples showed a clear increase in lipase/esterase production in samples containing polyethylene nanoparticles after 5 days of exposure. Clear zones of precipitation could be observed on selection agar around all samples tested after 24 hrs of growth as seen in Fig 4. Notably these changes were not seen in samples exposed to polyethylene microparticles or in a 0.1% Tween control (not pictured).

### Quantitative comparison of total RNA

**Fig 5:**
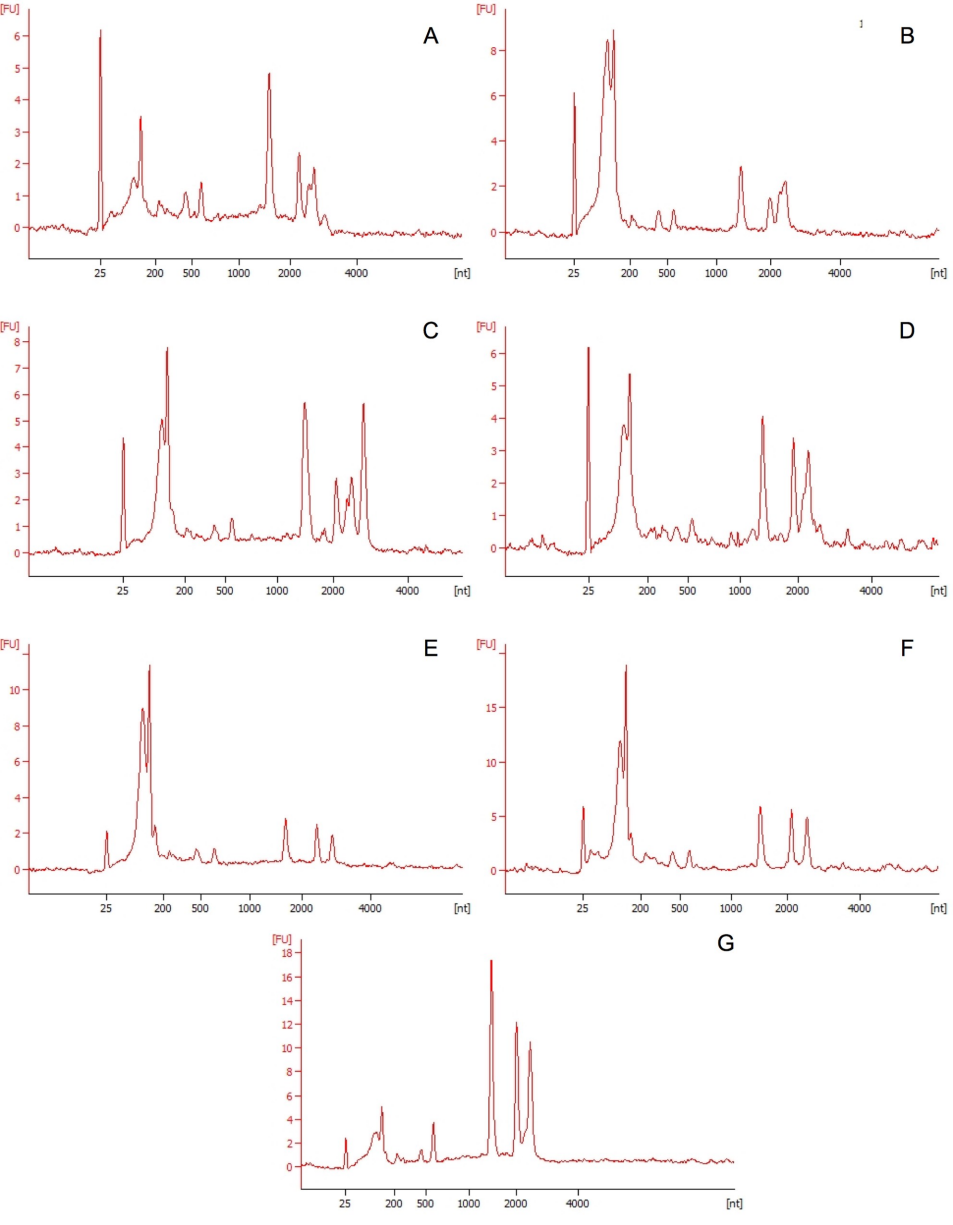
Electropherograms generated by Agilent RNA 6000. Electropherograms generated from total RNA extractions from *Synechococcus sp*. *7002* bacterial cells exposed to polyethylene microparticles and nanoparticles after 0, 5 and 10 days. A). Total RNA sample after 0 days exposure to polyethylene microparticles B) Total RNA sample after 0 days exposure to polyethylene nanoparticles C) Total RNA sample after 5 days exposure to polyethylene microparticles D) Total RNA sample after 5 days exposure to polyethylene nanoparticles. E) Total RNA sample after 10 days exposure to polyethylene microparticles F) Total RNA sample after 10 days exposure to polyethylene nanoparticles G) Control sample exposed to 0.1% Tween solution.

Extracted RNA was evaluated for RNA integrity by electrophoretic analysis on the Agilent Bioanalyzer 2100. Electropherograms from total RNA of samples exposed to microparticles or nanoparticles over a period of ten days can be seen in Fig 5. It should be noted that yields from RNA extractions of nanoparticle and microparticle exposed cells remained lower than that of the control sample. This is consistent with the literature, which shows lowered transcription in growth-limiting or stressful conditions. [14] Electropherograms also showed characteristic fragmentation of 23S rRNA a feature considered widespread in bacteria and influenced by cell age and culture conditions.

**Fig 6:**
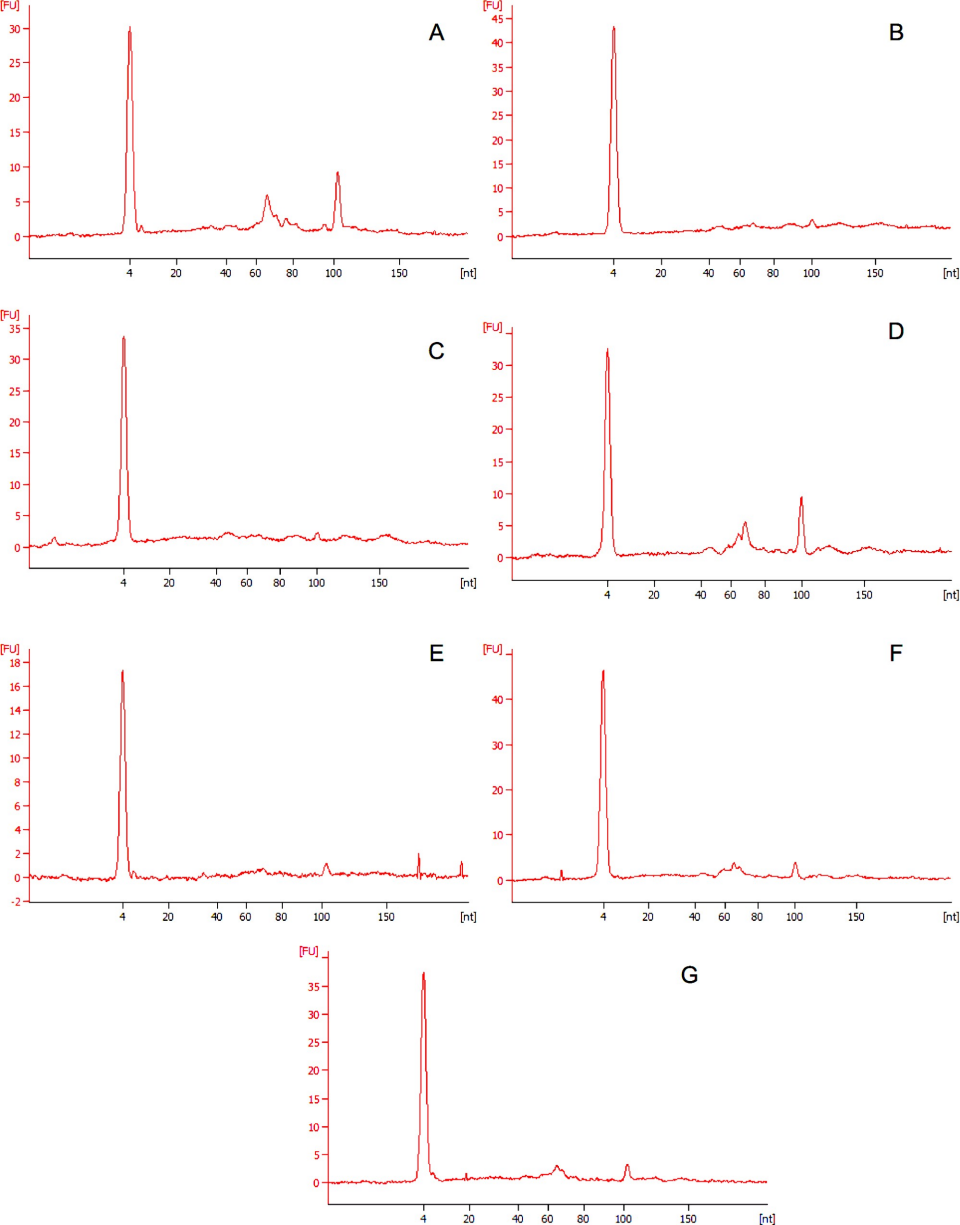
Electropherograms generated by Agilent RNA small. Electropherograms generated from total RNA extractions from *Synechococcus sp*. *7002* bacterial cells exposed to polyethylene microparticles after 0, 5 and 10 days. A). Small RNA sample after 0 days exposure to polyethylene nanoparticles B) Small RNA sample after 0 days exposure to polyethylene microparticles C) Small RNA sample after 5 days exposure to polyethylene nanoparticles D) Small RNA sample after 5 days exposure to polyethylene microparticles. E) Small RNA sample after 10 days exposure to polyethylene nanoparticles F) Small RNA sample after 10 days exposure to polyethylene microparticles G) Control sample exposed to 0.1% Tween solution.

Interestingly, electropherograms of cultures exposed to nanoparticles displayed lower relative intensities of both the 16S and 23S peaks than cultures exposed to microparticles or unexposed cultures. Decreases in 16S and 23S intensity have been shown in RNA profiles of hypoxic cultures within the literature and could be the result of increased cell stress. [29] To further quantify bacterial responses to stress a quantitation of small and microRNA was performed using the RNA Small kit. Electropherograms for microparticle and nanoparticle samples can be seen in Fig 6. miRNA ratios increased throughout exposure for microparticle samples and were significantly higher than that of control and nanoparticle samples at day 10 (41.5 ± 2.65, p< 0.01). Small RNA amounts were consistently lower among cultures exposed to nanoparticles over 5 and 10 day periods.

### Response to plastic size

**Fig 7:**
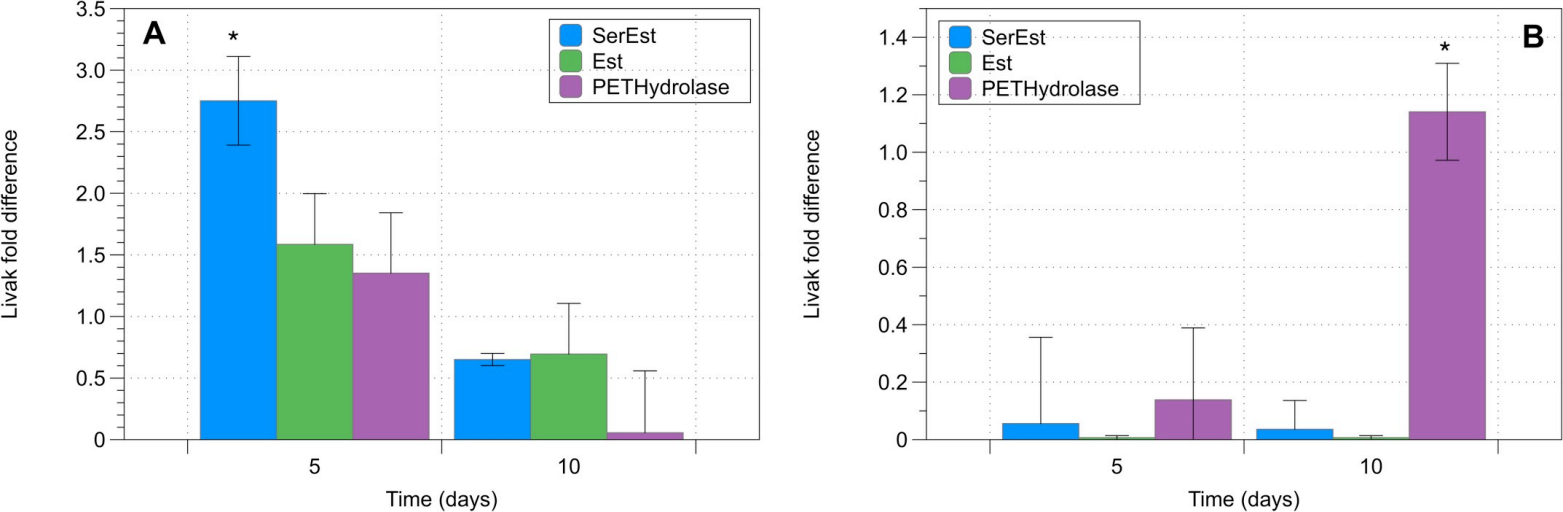
Comparison of transcript expression of selected hydrolase and lipase genes as measured by RT-PCR analysis of samples exposed to polyethylene particles. Normalized fold expression of selected genes relative to a control sample (time point = day 0). All genes were analyzed in triplicate by RT-PCR. Relative Quantification was carried out using the 2^−ΔΔCT^ method. A) Expression analysis for polyethylene nanoparticle samples B) Expression analysis for polyethylene microparticle samples. Data = mean +/SEM; N = 3; *p < 0.05 **p <0.01.

**Fig 8:**
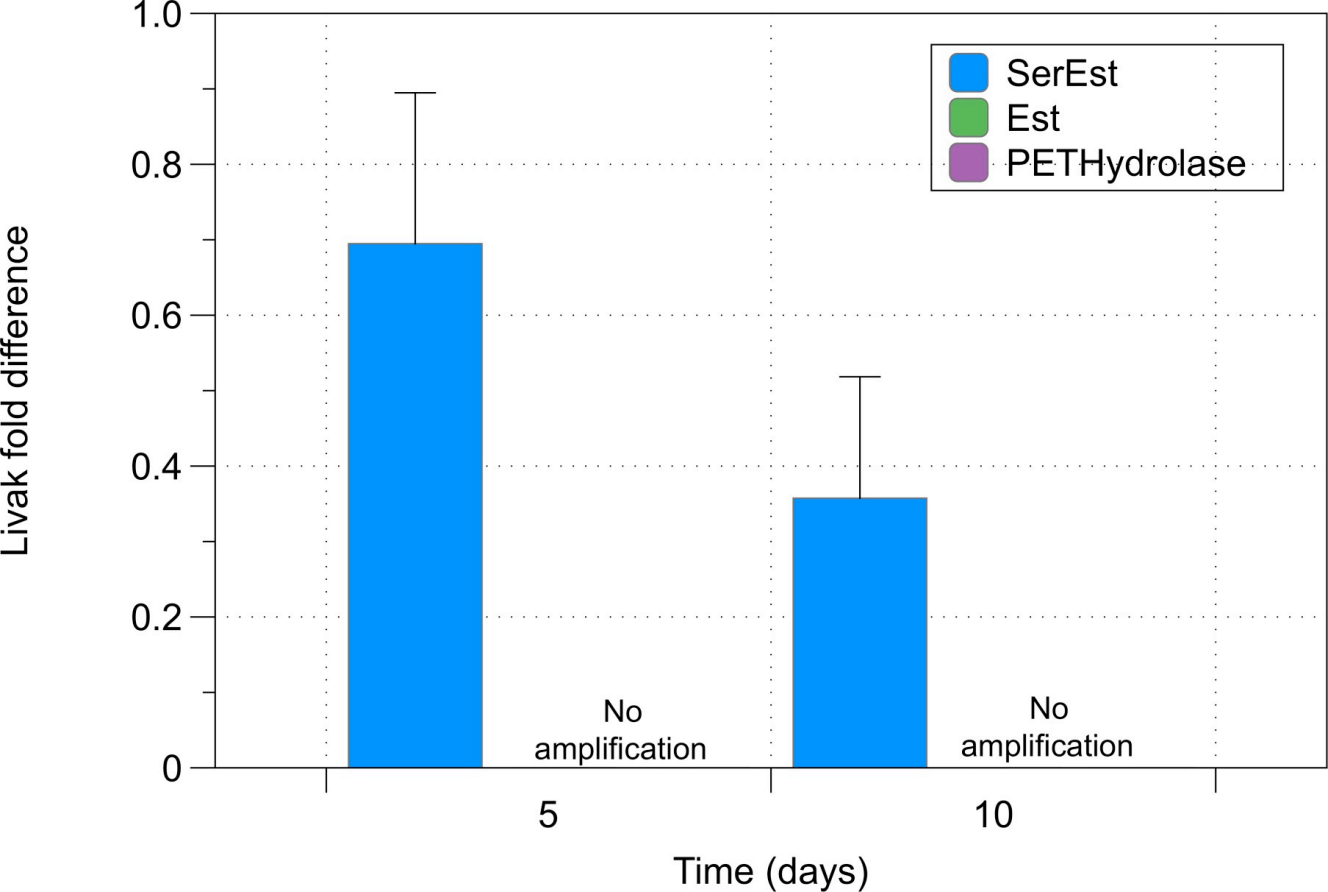
Transcript expression of control samples. Normalized fold expression of selected genes relative to a control sample (time point = day 0). All genes were analyzed in triplicate by RT-PCR. Relative Quantification was carried out using the 2^−ΔΔCT^ method. Data = mean +/SEM; N = 3.

Cultures of *Synechococcus elongatus pcc 7002* exposed to plastic particles showed increased expression of selected hydrolase and esterase genes with increasing time of incubation. Results of these experiments can be seen in Fig 7. Expression of hydrolases and lipases varied between microparticle and nanoparticle samples. As seen in Fig 7A, significant increases in all esterase genes could be seen at 5 days of exposure in the nanoparticle samples. Unlike nanoparticle samples, microparticle samples showed little change in expression of the selected genes at 5 days of exposure to particles. Increases in the expression of PETHydrolase were seen at day 10 in these samples. Results of control samples can be seen in Fig 8. An added analysis of a p20 domain protein (metacaspase) showed increased expression of this marker in nanoparticle samples after 5 days of exposure. (C_t_ = 28.0).

## Discussion

Synthetic plastics remain one of the key ecological stresses on the marine environment. Despite this, interactions between plastic and marine microorganisms are still poorly understood. This study reports for the first time the molecular responses of a widely recognized hydrocarbon degrading bacteria, *Synechococcus elongatus pcc 7002*, to polyethylene nanoparticles and microparticles. To our knowledge this study represents the first characterization of variations in total RNA and RNA expression in marine bacteria after exposure to polyethylene nanoparticles and microparticles.

Results of this investigation suggest that particle size and exposure time have significant effects on the degradation of stable RNA within marine bacteria. Especially at the nanoscale, these results suggest polyethylene particles have significant effects on the growth of *Synechococcus sp*. *7002*. Nanoparticles have a high surface to area ratio, which allows them to interact with the cell membrane. In prokaryotic cells the physiological functions of the cell are known to occur in different parts of the cell membrane and extensive breakdown of cellular RNA has been shown to occur when cells are exposed to membrane damaging reagents. [30] Nanoparticles, even particles not commonly bactericidal such as Au, have been shown to have similar effects on the bacterial cell, preventing the association of tRNA with the ribosome and affecting the cell membrane. [31] These effects could explain the large reduction in stable RNA at day 5 that occurs within the nanoparticle samples.

Our findings are consistent with the emerging literature on nanoplastic particles in the marine environment which has shown that nanoplastic particles are more toxic to small freshwater animals and zooplankton that microplastics. [19], [32] Sun et al. found that nanoparticle toxicity toward the marine bacterium *Halomonas alkaliphila* was closely correlated with particle size. [33] Their study also found increased EPS production within these cultures as a protective mechanism toward polystyrene nanoparticles. [33] Our investigations showed similar results with increased cell death and stable RNA degradation upon short exposures to nanoparticles and bacterial attachment and EPS formation at longer time points.

Qualitative and quantitative analysis of *Synechococcus sp*. *7002* exposed to nanoparticles and microparticles showed increased esterase production at 5 days and 10 days of culture respectively. Polyethylene degradation has been seen in a number of microorganisms within the literature. [34] *Synechococcus sp*. *7002* is an important hydrocarbon degrader in marine ecosystems, a trait shown to be associated with petroleum based plastic degradation and removal. [35] The environmental impacts and fates of plastics in the environment are still not well understood. Future analyses should expand upon these results by characterizing the full transcriptome of *Synechococcus sp*. *7002* exposed to these conditions and characterizing the lipases and esterases produced by these organisms.

## Conclusions

These results of this investigation demonstrate that exposure of marine bacteria such as *Synechococcus elongatus pcc 7002* to polyethylene microparticles and nanoparticles constitutes a significant stressor which influences not only gene expression but cell morphology and viability.

The data from this paper suggests that microplastics and nanoplastics could be a key source of microbial stress and may affect the molecular and enzymatic analysis of microbes in marine systems. These results argue that a better understanding of the biochemical characterization of microorganisms in marine systems affected by microplastics may be achieved by the further study of the changes in lipase and esterase gene expression.

## Supporting information

S1 Table Hemocytometer cell counts over seven day exposure to polyethylene nanoparticles and microparticles. (XLSX)

S1 Fig RT-PCR amplification curves for esterase genes after exposure to polyethylene nanoparticles and microparticles. (PPTX)
