# Supplementary material for: The response of Synechococcus sp. PCC 7002 to micro-/nano polyethylene particles - Investigation of a key anthropogenic stressor
Source: PLoS One. 2020 Jul 1;15(7):e0232745. doi: 10.1371/journal.pone.0232745 (PMC7329024; doi:10.1371/journal.pone.0232745)
Supplement: S1 Fig — (PPTX) [file pone.0232745.s002.pptx]

## Slide 1
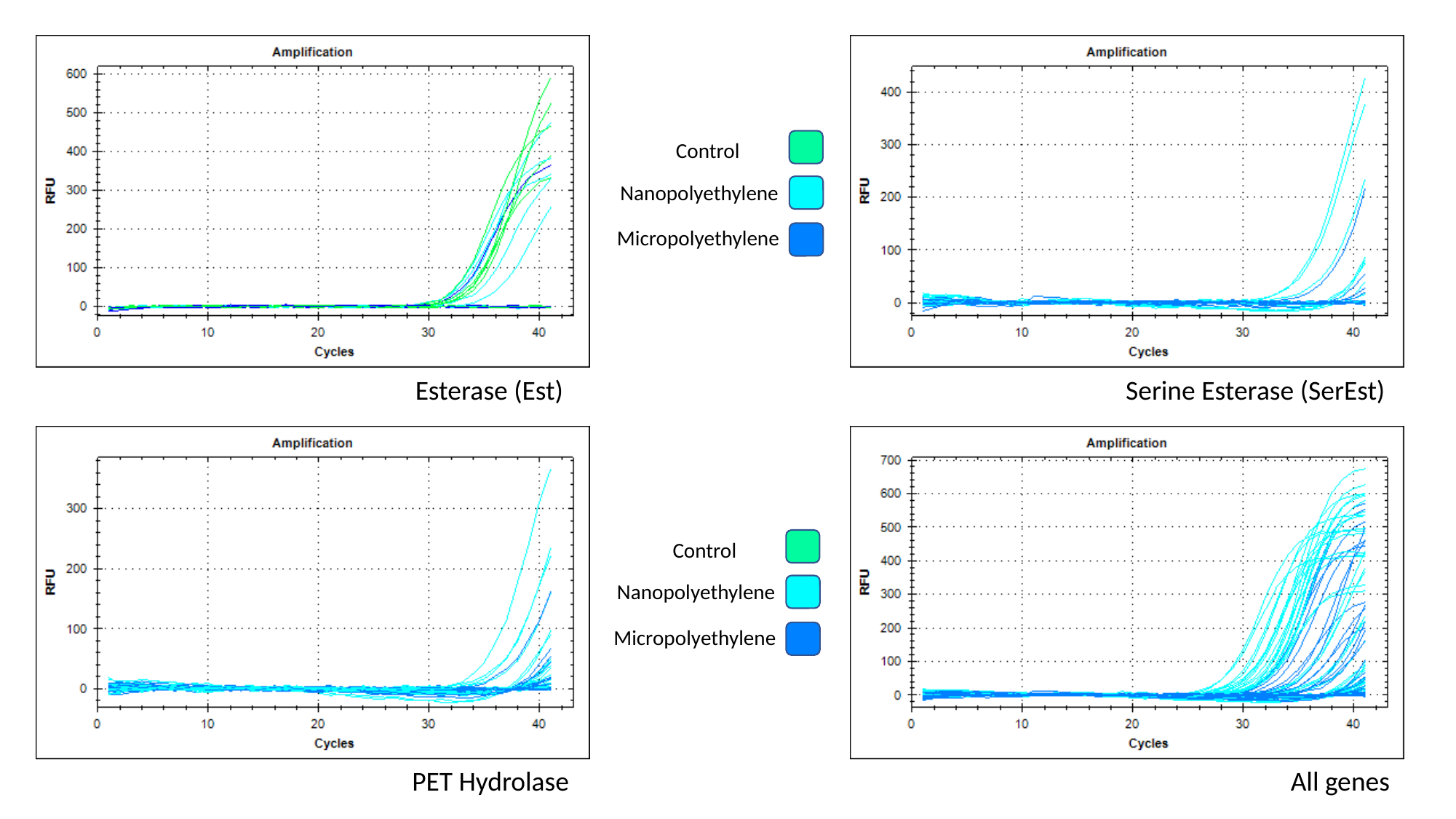

Control
Nanopolyethylene
Micropolyethylene
Esterase (Est)
Serine Esterase (SerEst)
Control
Nanopolyethylene
Micropolyethylene
PET Hydrolase
All genes
